# The Triumph of a New Idea

**Published:** 1907-02

**Authors:** 


### THE TRIUMPH OF A NEW IDEA.

It has been a subject of comment that the usual features of the Sunday newspapers showed too little variety. A recent departure in Sunday journalism has met with popular recognition and approval. The great illustrated weeklies and monthlies no longer have a monopoly of the periodical field.

Conan Doyle received $25,000 for the American serial rights of his last story, the highest price ever paid for similar rights. Anthony Hope, Jack London, Sewell Ford and many other popular novelists contribute to the publication which set the pace by paying this record price. Celebrated men and women write constantly for it on all subjects of timely interest. Clever verse, wit, humor and interesting miscellany complete a most interesting table of contents. It is profusely illustrated by the leading artists. In fact, the Sunday Magazine of the *Record-Herald* maintains the highest standard of periodical literature throughout. It gets the best at whatever cost.

